# A linear discriminant analysis model of imbalanced associative learning in the mushroom body compartment

**DOI:** 10.1371/journal.pcbi.1010864

**Published:** 2023-02-06

**Authors:** David Lipshutz, Aneesh Kashalikar, Shiva Farashahi, Dmitri B. Chklovskii

**Affiliations:** 1 Center for Computational Neuroscience, Flatiron Institute, New York, New York, United States of America; 2 Neuroscience Institute, New York University School of Medicine, New York, New York, United States of America; National Research Council, ITALY

## Abstract

To adapt to their environments, animals learn associations between sensory stimuli and unconditioned stimuli. In invertebrates, olfactory associative learning primarily occurs in the mushroom body, which is segregated into separate compartments. Within each compartment, Kenyon cells (KCs) encoding sparse odor representations project onto mushroom body output neurons (MBONs) whose outputs guide behavior. Associated with each compartment is a dopamine neuron (DAN) that modulates plasticity of the KC-MBON synapses within the compartment. Interestingly, DAN-induced plasticity of the KC-MBON synapse is imbalanced in the sense that it only weakens the synapse and is temporally sparse. We propose a normative mechanistic model of the MBON as a linear discriminant analysis (LDA) classifier that predicts the presence of an unconditioned stimulus (class identity) given a KC odor representation (feature vector). Starting from a principled LDA objective function and under the assumption of temporally sparse DAN activity, we derive an online algorithm which maps onto the mushroom body compartment. Our model accounts for the imbalanced learning at the KC-MBON synapse and makes testable predictions that provide clear contrasts with existing models.

## Author summary

To adapt to their environments, animals learn associations between sensory stimuli (e.g., odors) and unconditioned stimuli (e.g., sugar or heat). In flies and other insects, olfactory associative learning primarily occurs in a brain region called the mushroom body, which is partitioned into multiple compartments. Within a compartment, neurons that represent odors synapse onto neurons that guide behavior. The strength of these synapses is modulated by a dopamine neuron that responds to one type of unconditioned stimuli (e.g., sugar), which implicates these synapses as a biological substrate for associative learning in insects. Modifications of these synapses is imbalanced in the sense that dopamine-induced modifications only weaken the synapses and are temporally sparse. In this work, we propose a simple mechanistic model of learning in the mushroom body that accounts for this imbalanced learning. Our model is interpretable as implementing an algorithm for linear discriminant analysis, a classical statistical method for linearly separating feature vectors that belong to different classes. Our model makes testable predictions that provide clear contrasts with existing models.

## Introduction

Behavioral responses of animals are shaped in part by learned associations between sensory stimuli (e.g., odors) and unconditioned stimuli (e.g., sugar, heat or electric shock). A challenge in neuroscience is to understand the neural mechanisms that underlie associative learning. In invertebrates, the mushroom body is a well-studied brain region that plays a central role in olfactory associative learning [1–4]. The goal of this work is to propose a normative, mechanistic model of associative learning in the mushroom body that accounts for experimental observations and provides clear contrasts with existing models.

**Fig 1:**
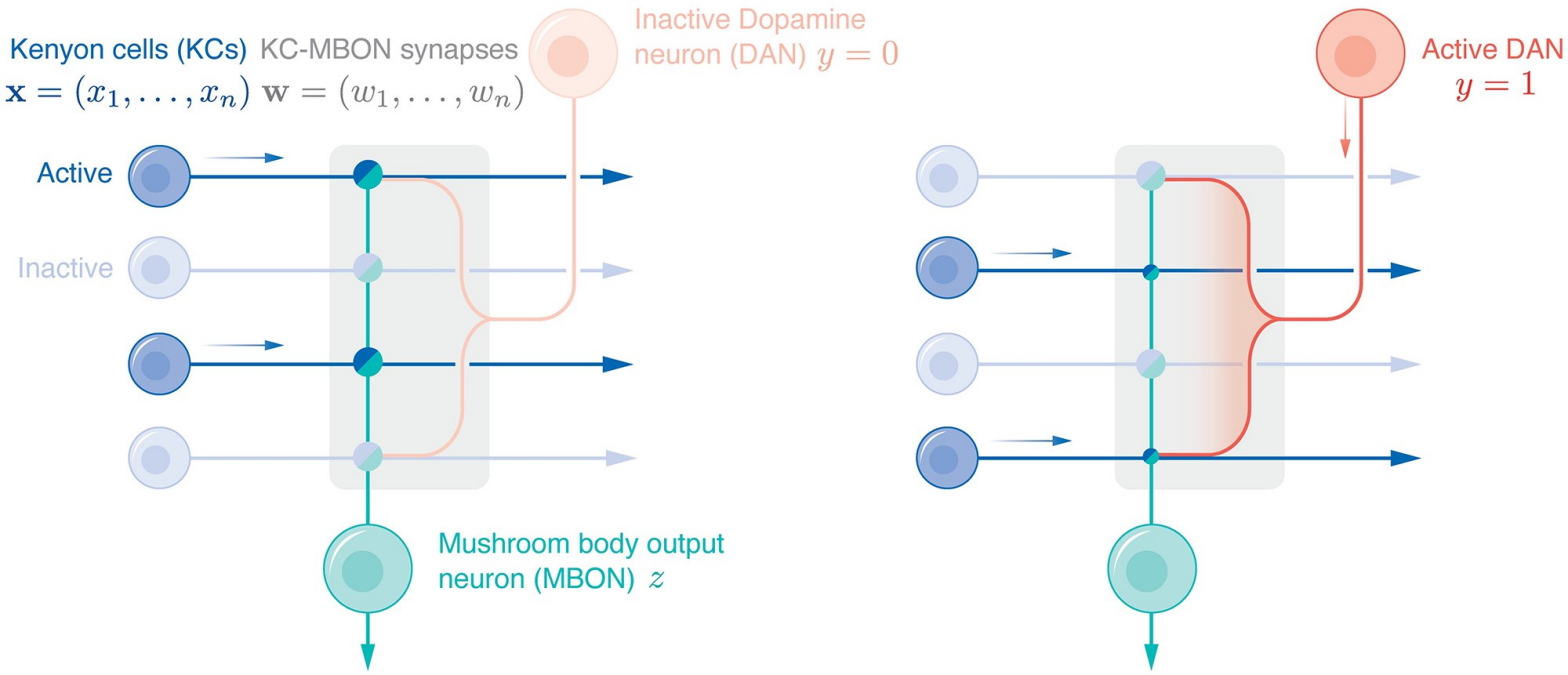
A simplified schematic of a mushroom body compartment. In both the left and right panels, the mushroom body compartment, indicated by the shaded box, is innervated by the axons of multiple KCs, the dendrites from one MBON and the axon terminals of one DAN. The bi-colored circles at the intersections of the KC axons and the MBON dendrites denote the KC-MBON synapses. Faintly shaded cell bodies indicate inactive neurons and boldly shaded cell bodies indicate active neurons. In the left panel, the DAN is inactive. In the right panel, the DAN is active and co-activation of the KCs and the DAN weakens the associated KC-MBON synapses (as illustrated by the smaller synapses).

The mushroom body is segregated into functionally independent compartments [5], Fig 1. Within each compartment, Kenyon cells (KCs), which encode sparse odor representations [6], form synapses with the dendrites of mushroom body output neurons (MBONs), whose outputs guide learned behavior [7]. Associated with each compartment is a single Dopamine neuron (DAN) that responds to an unconditioned stimulus [8, 9], and projects its axon into the mushroom body compartment where it innervates the KC-MBON synapses to modulate plasticity, implicating the KC-MBON synapse as the synaptic substrate for associative learning in invertebrates.

Experimental evidence suggests that learning at the KC-MBON synapse is imbalanced in the sense that DAN-induced plasticity is one-sided and temporally sparse. In particular, co-activation of a KC and the DAN *weakens* the KC-MBON synapse (see Fig 1, right) and DAN-induced plasticity is independent of the MBON activity [5]. This suggests that DAN-induced plasticity is one-sided and another mechanism such as homeostatic plasticity is responsible for strengthening the KC-MBON synapse. Furthermore, since each DAN responds to one type of unconditioned stimulus [2], which only constitutes a small fraction of all stimuli, the DAN activity is temporally sparse.

**Fig 2:**
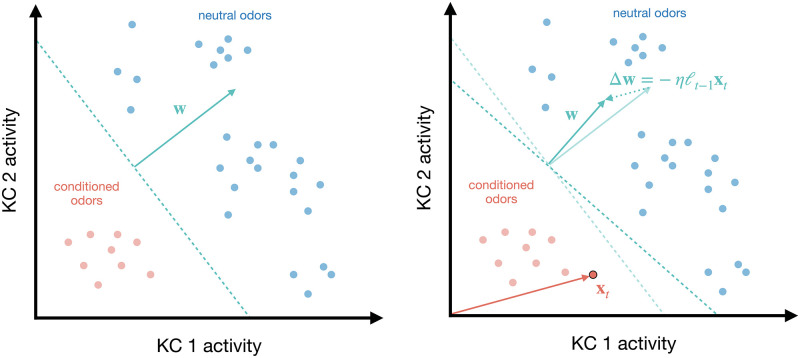
Linear separation of odors in the space of KC activities. Left: Illustration of the hyperplane H={x:w·x=b} (dashed teal line) in the space of KC activities that separates conditioned odor responses from neutral odor responses. Each light red (resp. blue) dot denotes the KC response to a conditioned (resp. neutral) odor. The teal arrow denotes the vector of KC-MBON synaptic weights **w**, which is translated to show that it is orthogonal to the hyperplane H. Right: Co-activation of the KCs and the DAN weakens the synaptic weights **w**. The KC activities **x**_*t*_ are denoted by the dark red dot with black border. The change of the synaptic weights Δ**w** is in the direction −**x**_*t*_. The hyperplane H rotates to remain orthogonal to **w**. The change in bias Δ*b*, which translates the hyperplane, is not depicted.

In this work, we propose a normative, mechanistic model of associative learning in the mushroom body that accounts for the imbalanced learning. We model each MBON as a linear discriminant analysis (LDA) classifier, which predicts if an associated unconditioned stimulus is present (the class label) given a KC odor representation (the feature vector). Under this interpretation, the KC-MBON synapses and an MBON bias term define a hyperplane in the high-dimensional space of KC odor representations that separates odor representations associated with the unconditioned stimulus from all other odor representations, Fig 2.

Here, ‘normative’ refers to the fact that our mechanistic model is interpretable as an algorithm for optimizing an LDA objective. The normative approach is top-down in the sense that first the circuit objective is proposed and then an optimization algorithm is derived and compared with known physiology. There are several advantages to this approach. First, it directly relates the circuit objective to its mechanism; for example, neural activities and synaptic weight updates are interpretable as steps in an algorithm for solving a relevant circuit objective. Second, the approach distills down what aspects of the physiological are essential for optimizing the circuit objective and what aspects are not captured by the objective. Third, normative models are often analytically tractable, which allows them to be analyzed for any input statistics without resorting to exhaustive numerical simulation.

To derive our algorithm, we start with a convex objective for LDA (in terms of the KC-MBON synaptic weights). The objective can be optimized in the offline setting by taking gradient descent steps with respect to the KC-MBON synaptic weights. To obtain an online algorithm that accounts for the imbalanced learning, we take advantage of the fact that DAN activity is temporally sparse to obtain online approximations of the input statistics. Finally, we show numerically that our algorithm performs well even when DAN activity is not temporally sparse.

Our model makes testable predictions that are a direct result of the learning imbalance. First, our model predicts that DAN-induced plasticity at the KC-MBON synapse is sensitive to the time elapsed since the DAN was last active. Second, our model predicts that if the DAN is never active, then the KC-MBON synapses adapt to align with the mean KC activity (normalized by the covariance of the KC activity).

## Results

### LDA model of the mushroom body compartment

We consider a simplified mushroom body compartment that consists of *n* KC axons, the axon terminals from one DAN and the dendrites of one MBON, Fig 1. At each time *t* = 1, 2, …, the vector $x_{t}\in R^{n}$ encodes the KC activities and the scalar *y*_*t*_ ∈ {0, 1} indicates whether the DAN is active (*y*_*t*_ = 1) or inactive (*y*_*t*_ = 0). If the DAN is active, we refer to **x**_*t*_ as a *conditioned odor response*, whereas if the DAN is inactive, we refer to **x**_*t*_ as a *neutral odor response*. We assume the DAN activity is temporally sparse, which can be expressed mathematically as *π*_1_ ≪ 1, where *π*_1_ ≔ 〈*y*_*t*_〉_*t*_ is the fraction of time that the DAN is active.

In our model, the MBON is a linear classifier that predicts the DAN activity *y*_*t*_ (class label) given the KC activities **x**_*t*_ (feature vector). Let $w\in R^{n}$ be a synaptic weight vector whose *i*^th^ component represents the strength of the synapse between the *i*^th^ KC and the MBON. At each time *t*, the KC activities **x**_*t*_ are multiplied by the synaptic weight vector **w** to generate the total input to the MBON, denoted *c_t_* ≔ **w** · **x**_*t*_. The output (firing rate) of the MBON is given by

$$\begin{array}{c} z_{t}≔\text{max}\left(c_{t}-b,0\right), \end{array}$$

where *b* represents the ‘bias’ of the MBON; that is, the threshold below which the MBON does not fire. Under this interpretation, the KC-MBON synapses **w** and MBON bias *b* define a hyperplane $H:=\{x\in R^{n}:w\cdot x=b\}$ in the *n*-dimensional space of KC activities that separates conditioned odor responses from neutral odor responses, Fig 2. In this case, *z*_*t*_ > 0 (resp. *z*_*t*_ = 0) corresponds to the prediction *y*_*t*_ = 0 (resp. *y*_*t*_ = 1). In other words, the MBON is a linear classifier that is active when predicting there is no unconditioned stimulus and inactive when predicting there is an unconditioned stimulus, which is consistent with experimental observations [2].

We derive learning rules for the KC-MBON synaptic weights **w** (and bias *b*) that solve an LDA objective and are consistent with experimental observations [2, 5]. LDA is popular linear classification method that is optimal under the assumption that the neutral odor responses and conditioned odor responses are Gaussian with common covariance matrix, but works well in practice even when these assumptions do not hold [10].

Our starting point is the convex LDA objective

$$\begin{array}{ccc} \underset{w}{\text{min}}L\left(w\right), & & L\left(w\right)≔-w\cdot \left(\mu_{0}-\mu_{1}\right)+\frac{1}{2}w^{⊤}\Sigma w, \end{array}$$ (1)

where ***μ***_0_ and ***μ***_1_ denote the means of the neutral odor responses and conditioned odor responses, respectively, and **Σ** denotes the covariance of the neutral odor response. In the offline setting, we can minimize *L*(**w**) by taking gradient steps with respect to **w**:

$$\begin{array}{c} \Delta w=\eta (\mu_{0}-\mu_{1}-\Sigma w), \end{array}$$ (2)

where *η* > 0 is the step size. However, computing the means ***μ***_0_, ***μ***_1_ and covariance **Σ** requires the MBON to have access to the entire sequence of inputs, which is an unrealistic assumption.

To derive our online algorithm, we replace the averages ***μ***_0_, ***μ***_1_ and **Σ** in Eq 2 with online estimates. When the DAN is inactive (*y*_*t*_ = 0), we update the KC-MBON weights **w** according to the homeostatic plasticity rule

$$\begin{array}{c} y_{t}=0:\;\Delta w=\eta \left(\mu_{0,t}-\left(c_{t}-\zeta_{t}\right)\left(x_{t}-\mu_{0,t}\right)\right), \end{array}$$ (3)

where ***μ***_0,*t*_ denotes the running estimate of the mean neutral odor response and *ζ*_*t*_ denotes the running estimate of the mean total MBON input *c*_*t*_ conditioned on the DAN being inactive. Here, ***μ***_0,*t*_ and (*c_t_* − *ζ_t_*) (**x**_*t*_ − ***μ***_0,*t*_) are online estimates of ***μ***_0_ and **Σ**, respectively (see Methods section). The running means ***μ***_0,*t*_ and *ζ*_*t*_ can be represented by biophysical quantities such as calcium concentrations at the pre- and postsynaptic terminals of the KC-MBON synapses.

When the DAN is active, we update the KC-MBON weights **w** according to the following DAN-induced plasticity rule

$$\begin{array}{c} y_{t}=1:\;\Delta w=-\eta \ell_{t-1}x_{t}, \end{array}$$ (4)

where *ℓ*_*t*−1_ denotes the time elapsed since the last time the DAN was active; see Fig 2 (right) for a geometric interpretation of the plasticity rule. The update in Eq 4 is in line with experimental evidence showing that DAN-induced plasticity is independent of the MBON activity *z*_*t*_ and co-activation of the KCs and the DAN reduces the strength of the synapses between the KCs and the MBON [5]. Biologically, the scalar *ℓ*_*t*−1_ can be represented as the sensitivity of the KC-MBON synapses to DAN-induced plasticity. Assuming the conditioned odor response is independent of the time elapsed between DAN activations, then on average, the update in Eq 4 is approximately equal to −*η**μ***_1_, see Methods section. Therefore, the updates in Eqs 3–4 together account for all three terms in the offline update in Eq 2. The full model, including the bias updates (see Methods section), is summarized in Algorithm 1.

**Algorithm 1** LDA in the mushroom body compartment

**input:** (**x**_1_,*y*_1_), … ,(**x**_*T*_,*y*_*T*_)

**initialize:** **w** = (*w*_1_, … ,*w_n_*), *b* = 0, *ℓ*_0_ = 1, *η* > 0

**for** *t* = 1, 2, …, *T* **do**

 *c_t_* ← **w** · **x**_*t*_

 *z*_*t*_ ← max(*c*_*t*_ − *b*, 0)

 **if** *y*_*t*_ = 0 **then**

  $\mu_{0,t}\leftarrow \mu_{0,t-1}+\frac{1}{t}\left(x_{t}-\mu_{0,t-1}\right)$

  $\zeta_{t}\leftarrow \zeta_{t-1}+\frac{1}{t}\left(c_{t}-\zeta_{t-1}\right)$

  $b\leftarrow b+\frac{1}{t}\left(\frac{1}{2}c_{t}-b\right)$

  **w** ← **w** + *η*(***μ***_0,*t*_ − (*c_t_* − *ζ_t_*)(**x**_*t*_ − ***μ***_0,*t*_))

  *ℓ*_*t*_ ← *ℓ*_*t*−1_ + 1

 **else if** *y*_*t*_ = 1 **then**

  $b\leftarrow b+\frac{1}{t}\left(\frac{1}{2}\ell_{t-1}c_{t}-\text{log}\ell_{t-1}-b\right)$

  **w** ← **w** − *ηℓ*_*t*−1_**x**_*t*_

  *ℓ*_*t*_ ← 1

 **end if**

**end for**

Algorithm 1 only has one hyper-parameter—the learning rate *η* > 0—which corresponds to timescale for learning in the mushroom body compartment. Hige et al. [5] showed that mushroom body compartments have distinct timescales for learning, which can be modeled by choosing different learning rates *η* > 0.

### Numerical experiments

Next, we test Algorithm 1 on synthetic and real datasets. We test our algorithm on inputs when our assumption *π*_1_ ≪ 1 holds, but also on inputs when *π*_1_ ≈ 0.5. To evaluate our algorithm, we measure the running accuracy of the projections *z*_*t*_ over the previous min(100, *t*) iterations, where the algorithm is accurate at the *t*^th^ iterate if *z*_*t*_ = 0 and *y*_*t*_ = 1 or if *z*_*t*_ > 0 and *y*_*t*_ = 0.

#### Synthetic dataset

**Fig 3:**
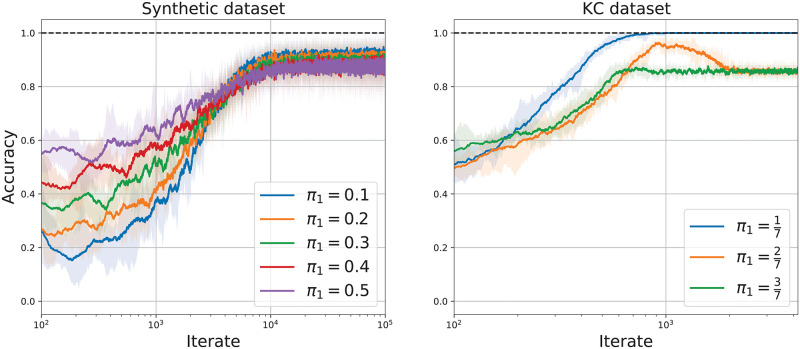
Performance of Algorithm 1. Accuracy of Algorithm 1 on the synthetic datasets (left) and the KC dataset (right). Each line denotes the mean accuracy over 10 runs. Each shaded region indicates the area between the minimum and maximum accuracy over 10 runs.

We begin by evaluating Algorithm 1 on a synthetic dataset generated by a mixture of 2 overlapping Gaussian distributions, so that the optimal accuracy is less than 1. The data points of the 2 classes are each drawn from a 2-dimensional mean with common covariance. We simulate datasets of 10^5^ data points using the same mean and covariance in both classes but vary the frequency of class 1 samples encountered. We consider the cases *π*_1_ = 0.1, 0.2, 0.3, 0.4, 0.5. In Fig 3 (left) we plot the error and the accuracy of our model for varying *π*_1_. Remarkably, while the derivation of Algorithm 1 relied on the fact that *π*_1_ ≪ 1, the algorithm still performs well even when *π*_1_ = 0.5.

#### KC activities dataset

We test our model on KC activities reported in [11]. Campbell et al. recorded odor-evoked KC responses in the fly mushroom body. The dataset we tested on contains the responses of 124 KCs in a single fly to the presentation of 7 odors, see [11, Figure 1]. To ensure the KC responses are well conditioned, we add Gaussian noise with covariance *ϵ* **I**_124_, where *ϵ* = 0.01. We apply Algorithm 1 to the KC dataset. We first consider the case that odor 1 denotes the class 1 odor and odors 2–7 denote the class 0 odors, so $\pi_{1}=\frac{1}{7}$. We then consider the cases that odors 1–2 (resp. 1–3) odors denote the class 1 odors and the remaining odors denote the class 0 odors, so $\pi_{1}=\frac{2}{7}$ (resp. $\pi_{1}=\frac{3}{7}$). In Fig 3 (right) we plot the error and accuracy of our model for varying *π*_1_. Impressively, the modified algorithm performs well (approximately 85% accuracy) even when the assumption *π*_1_ ≪ 1 is violated.

#### Competing MBONs

**Fig 4:**
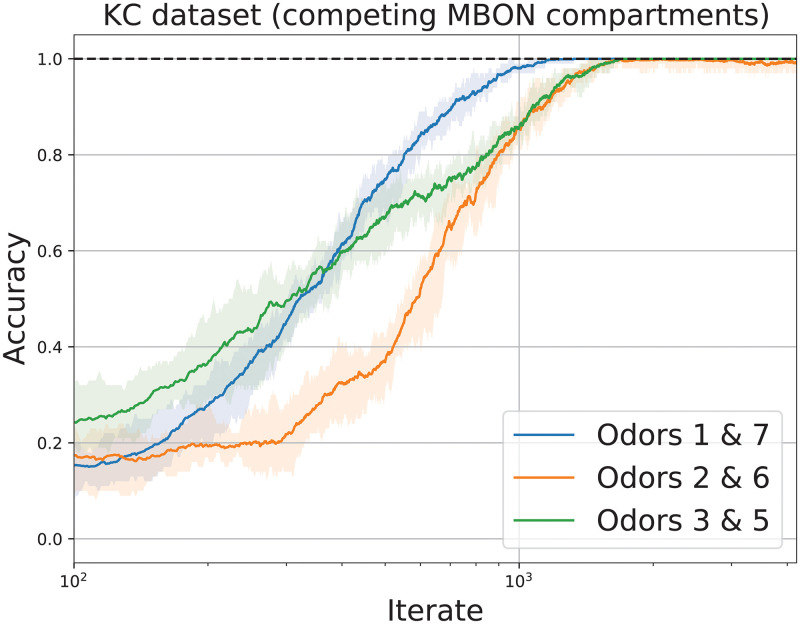
Performance of competing MBONs. Accuracy of 2 parallel runs of Algorithm 1 on the KC dataset to classify odors as aversive, attractive or neutral. Each line denotes the mean accuracy over 10 runs. Each shaded region indicates the area between the minimum and maximum accuracy over 10 runs.

Using the KC activities dataset, we model 2 MBONs with competing valences by running 2 instances of Algorithm 1 in parallel with different class assignments for the odors. We consider the case that odor 1 is aversive, odor 7 is attractive and the remaining odors are neutral. For MBON 1 (resp. MBON 2), we assume that odor 1 (resp. odor 7) denotes the class 1 odor and odors 2–7 (resp. odors 1–6) denote the class 0 odors, so that MBON 1 (resp. MBON 2) activity promotes approach (resp. avoid) behavior. Let *z*_*i*,*t*_ denote the output of MBON *i* ∈ {1, 2}. At each iterate *t*, if odor 1 (resp. odor 7, odors 2–6) is presented, then the model is accurate if *z*_1,*t*_ > 0 and *z*_2,*t*_ = 0 (resp. *z*_1,*t*_ = 0 and *z*_2,*t*_ > 0, resp. *z*_1,*t*_ > 0 and *z*_2,*t*_ > 0), and inaccurate otherwise. We then repeat the experiment two more times, but with odor 2 (resp. odor 3) labeled as aversive and odor 6 (resp. odor 5) as attractive. In Fig 4, we plot the performance of the competing MBONs.

## Discussion

### Summary

In this work, we proposed a normative model of the mushroom body compartment that accounts for imbalanced learning at the KC-MBON synapse. Testing our model on synthetic and real datasets shows that it performs well under a variety of conditions. In our model, DAN-induced plasticity at the KC-MBON synapse does not depend on the MBON activity, but rather on the time elapsed since the last time the DAN was active. This aspect of our model suggests testable predictions that provide clear contrasts with existing models of associative learning in the mushroom body.

### Model predictions

*Prediction 1*—In the absence of DAN activity, the KC-MBON synapses will align with the mean KC activity normalized by the covariance of their activities. When presented with neutral odors, the synapses adapt according to the homeostatic update in Eq 3. Since this update is equal to *η*(***μ***_0_ − **Σw**) on average (see Methods section), the KC-MBON synaptic weights equilibrate at **w** = **Σ**^−1^***μ***_0_. Experimentally, this prediction could be tested by first presenting a fly with neutral odors and simultaneously recording from multiple KCs and an MBON. The weights can be estimated from the neural activities (using, e.g., [12]) and compared with our prediction **w** = **Σ**^−1^***μ***_0_.

*Prediction 2*—DAN-induced plasticity is proportional to the time elapsed since the DAN was last active. According to update in Eq 4, DAN-induced plasticity is proportional to the time elapsed since the DAN was last active. Experimentally, this prediction could be tested by presenting a fly with conditioned odors with different time intervals between presentations and estimating the resulting change in the synaptic weights.

### Relation to existing models

There are a number of existing computational models of associative learning in the mushroom body [13–17], many of which are faithful to biophysical details and successfully capture important computational principles underlying associative learning in the mushroom body (see, e.g., [15]). Through extensive numerical simulations, these computational models can explain a number of phenomena. For example, Heurta and Nowotny [15] show that the organization of the mushroom body supports fast and robust associative learning, Bazhenov et al. [16] show that interactions between unsupervised and supervised forms of learning can explain how the timescale of associative learning depends on experimental conditions, and Peng and Chittka [17] show how complex forms of learning (e.g., peak shift) depend on different mechanistic aspects of learning in the mushroom body. In this work, we propose a top-down normative model of learning at the KC-MBON synapse, which contrasts with the bottom-up approach in these works that build models closely tied to physiological evidence. In this way, the our model is interpretable as an algorithm for optimizing a circuit objective and the output can be predicted analytically for any environmental condition without needing to resort to numerical simulation. In addition, our normative model makes testable predictions that are in clear contrast with these models, providing a method for validating or invalidating our model.

In addition to these models, Bennett et al. [18] propose a reinforcement learning model of the KC-MBON synapses as minimizing reinforcement prediction errors. They first consider a model in which the reinforcement signal is computed as the difference between DAN activities, so their plasticity rule requires 2 DANs to innervate a single mushroom body compartment, which is in contrast to experiment evidence showing that most compartments only receive inputs from a single DAN [9]. To account for this experimental observation, they propose a heuristic modification that adds a constant source of synaptic potentiation, which can be viewed as a form of homeostatic plasticity and is in line with experimental evidence. However, the modification is not normative and can fail to minimize prediction errors.

A significant difference between our model and these existing models is that DAN-induced plasticity depends on *ℓ*_*t*−1_, the time elapsed since the DAN was last active. In our model, the variable *ℓ*_*t*−1_ is critical for balancing homeostatic plasticity and DAN-induced plasticity. In S1 Appendix, we consider a modification of our algorithm in which *ℓ*_*t*−1_ is replaced by a fixed constant ℓ_*_.

### Comparison of LDA to other linear classification methods

LDA is a linear classifier that is optimal under strict assumptions on the inputs, so it is worth considering other linear classification methods such as logistic regression and support vector machines (SVMs). Logistic regression is classical method for estimating the probability of one class versus another class. In terms of performance, there is evidence that there is not a substantial difference in the performance of logistic regression and LDA even when the assumptions for LDA are not met [19]. As a model of the insect mushroom body, we are unaware of an online algorithm for logistic regression that maps onto the mushroom body compartment and matches the experimental observations in [2, 5].

SVMs are flexible linear classifiers that do not make assumptions about the underlying data distribution. Huerta et al. [13, 15] proposed models of the mushroom body that are closely related to SVMs [20, 21]; however, the DAN-induced synaptic update rules depend on the MBON activity, which is in contrast to recent experimental evidence [5].

### Limitations

Our model is a dramatic simplification of the mushroom body focused on providing a normative account of learning at the KC-MBON synapse that can account for how balance between DAN-induced plasticity and homeostatic plasticity is optimally maintained. Consequently, our model does not account for a number of the physiological details. For example, in order to implement an LDA algorithm, we do not sign-constrain the synaptic weight vector **w**, which violates Dale’s law. In addition, we assume that the DAN activity is binary. In reality the DAN may fire at different rates depending on the strength of the unconditioned stimulus and the firing rate may affect the DAN-induced plasticity. We can modify our model to allow *y*_*t*_ to be any nonnegative scalar and replace the update in Eq 4 with Δ**w** = −*ηℓ*_*t*−1_*y_t_* **x**_*t*_. However, in this case the algorithm is not derived from an objective function for LDA and so it is more challenging to understand the output. In addition to such simplifications, there are other features such as feedback connections in the mushroom body that have been recently discovered and are relevant for associative learning [7, 22], which are also not captured by our model.

## Methods

### Linear discriminant analysis

LDA is a statistical method for linear classification [23, section 4.3], which makes the following simplifying assumption: the conditional probability distributions *p*(**x**|*y* = 0) and *p*(**x**|*y* = 1) are both Gaussian with common full-rank *n* × *n* covariance matrix **Σ**; that is

$$\begin{array}{ccc} p\left(x|y=0\right)∼N\left(\mu_{0},\Sigma \right), & & p\left(x|y=1\right)∼N\left(\mu_{1},\Sigma \right), \end{array}$$

where ***μ***_0_ and ***μ***_1_ denote the means of the class 0 and class 1 feature vectors. In this case, the optimal decision criteria for assigning class 0 (resp. class 1) to feature vector **x** is **w** · **x** > *b* (resp. **w** · **x** < *b*), where

$$\begin{array}{ccc} w_{\text{opt}}:=\Sigma^{-1}\left(\mu_{0}-\mu_{1}\right), & & b_{\text{opt}}:=\frac{1}{2}w\cdot \left(\mu_{0}+\mu_{1}\right)+\text{log}\frac{\pi_{1}}{\pi_{0}}, \end{array}$$ (5)

and *π*_*i*_ denotes the probability that a samples belongs to class *i*, for *i* = 0, 1. In particular, the hyperplane $H=\{x\in R^{n}:w_{\text{opt}}\cdot x=b_{\text{opt}}\}$ defines the optimal separation boundary for predicting whether a feature vector belongs to class 0 or class 1. While LDA assumes a specific generative model, it performs well in practice even when the assumptions do not hold [10].

The optimal weights **w**_opt_ can be expressed as the solution of the convex minimization problem in Eq 1, which we can solve for by taking the gradient descent steps (Eq 2). Formally, taking the step size *η* to zero in Eq 2 yields the linear gradient flow

$$\begin{array}{c} \dot{w}\left(t\right)=\mu_{0}-\mu_{1}-\Sigma w\left(t\right), \end{array}$$

whose solution is given by

$$\begin{array}{c} w\left(t\right)=e^{-\Sigma t}w\left(0\right)+(I_{n}-e^{-\Sigma t})\Sigma^{-1}\left(\mu_{0}-\mu_{1}\right). \end{array}$$

In particular, we see that the solution **w**(*t*) converges exponentially to the optimal solution **w**_opt_.

### An online algorithm for imbalanced learning

In the online setting, the class means ***μ***_0_, ***μ***_1_ and the covariance **Σ** are not available. Instead, at each time *t* the algorithm has access to the feature vector **x**_*t*_ and class label *y*_*t*_. To derive our online algorithm, we make online approximations of the offline quantities ***μ***_0_, ***μ***_1_ and **Σ** that are based on the fact that the unconditioned stimuli are sparse in time, i.e., *π*_1_ ≪ 1, where we recall that *π*_1_ denotes the proportion of conditioned odors. First, we note that we can rewrite the sample class means

$$\begin{array}{cccc} \mu_{0} & =\frac{1}{\pi_{0}}{\left\langle \left(1-y_{t}\right)x_{t}\right\rangle }_{t}, & & \mu_{1}=\frac{1}{\pi_{1}}{\left\langle y_{t}x_{t}\right\rangle }_{t}, \end{array}$$

where *π*_0_ ≔ 〈1 − *y*_*t*_〉_*t*_ ≈ 1 is the fraction of odors that are neutral, and the sample covariance

$$\begin{array}{cc} \Sigma & ={\left\langle \left(1-y_{t}\right)\left(x_{t}-\mu_{0}\right){\left(x_{t}-\mu_{0}\right)}^{⊤}\right\rangle }_{t}+{\left\langle y_{t}\left(x_{t}-\mu_{1}\right){\left(x_{t}-\mu_{1}\right)}^{⊤}\right\rangle }_{t}. \end{array}$$

#### Estimating the mean response to a neutral odor and the covariance

Since *π*_0_ ≈ 1, we approximate

$$\begin{array}{ccc} \mu_{0}\approx {\left\langle \left(1-y_{t}\right)x_{t}\right\rangle }_{t}, & & \Sigma \approx {\left\langle \left(1-y_{t}\right)\left(x_{t}-\mu_{0}\right){\left(x_{t}-\mu_{0}\right)}^{⊤}\right\rangle }_{t}. \end{array}$$ (6)

Therefore, in the online setting, we can keep a running estimate of ***μ***_0_ and *ζ* ≔ **w** · ***μ***_0_ ≈ 〈(1 − *y_t_*)*c_t_*〉_t_, where we recall that *c_t_* = **w** · **x**_*t*_, by performing the updates

$$\begin{array}{ccc} \mu_{0,t}\leftarrow \mu_{0,t-1}+\frac{1}{t}\left(1-y_{t}\right)\left(x_{t}-\mu_{0,t-1}\right), & & \zeta_{t}\leftarrow \zeta_{t-1}+\frac{1}{t}\left(1-y_{t}\right)\left(c_{t}-\zeta_{t-1}\right). \end{array}$$ (7)

In view of Eq 6 and the definitions of *c*_*t*_ and *ζ*, we can replace **Σw** with the online approximation

$$\begin{array}{c} \left(1-y_{t}\right)\left(x_{t}-\mu_{0}\right){\left(x_{t}-\mu_{0}\right)}^{⊤}w=\left(1-y_{t}\right)\left(c_{t}-\zeta_{t}\right)\left(x_{t}-\mu_{0,t}\right). \end{array}$$ (8)

We replace the first and third terms in the offline update in Eq 2, *η*(***μ***_0_ − **Σw**), with the online estimate *η*(1 − *y_t_*)(***μ***_0,*t*_ − (*c_t_* − *ζ_t_*)(**x**_*t*_ − ***μ***_0,*t*_)).

#### Estimating the mean response to a conditioned odor

To obtain an online approximation of ***μ***_1_, we first note that $\frac{1}{\pi_{1}}$ is approximately equal to the average time elapsed between class 1 samples. To see this, let *t*_1_, *t*_2_, … denote the subset of times such that *y*_*t*_ = 1. Then, letting *t*_0_ = 0, we have

$$\begin{array}{c} \frac{1}{\pi_{1}}=\underset{r\to \infty }{\text{lim}}\frac{t_{r}}{r}=\underset{r\to \infty }{\text{lim}}\frac{1}{r}\sum_{j=1}^{r}\left(t_{j}-t_{j-1}\right). \end{array}$$

Thus, in the online setting, when the *j*^th^ class 1 sample is presented (i.e., *y*_*t*_ = 1), we can use the time elapsed since the last class 1 sample, *t*_*j*_ − *t*_*j*−1_, as an online estimate of $\frac{1}{\pi_{1}}$. Setting *ℓ*_0_ = 1 and

$$\begin{array}{c} \ell_{t}=\{\begin{array}{cc} \ell_{t-1}+1 & \text{if}\;y_{t}=0\\ 1 & \text{if}\;y_{t}=1, \end{array} \end{array}$$

we see that at time *t* such that *y*_*t*_ = 1, *ℓ*_*t*−1_ denotes the time elapsed since the last class 1 sample, so ${\langle \ell_{t-1}|y_{t}=1\rangle }_{t}=\frac{1}{\pi_{1}}$. Assuming that the variables *ℓ*_*t*−1_ and **x**_*t*_ are independent given *y*_*t*_ = 1—i.e., the KC representation **x**_*t*_ of a conditioned odor is independent of the time elapsed since the last conditioned odor *ℓ*_*t*−1_—we see that ***μ***_1_ = 〈*ℓ*_*t*−1_|*y_t_* = 1〉_*t*_ 〈**x**_*t*_|*y_t_* = 1〉_*t*_ = 〈*ℓ*_*t*−1_**x**_*t*_|*y_t_* = 1〉_*t*_. We replace the second term in the offline update in Eq 2, −*η**μ***_1_, with the online approximation −*ηy*_*t*_*ℓ*_*t*-1_**x**_*t*_.

#### Estimating the bias

To estimate the bias *b*, we note that because *π*_0_ ≈ 1 and $\frac{1}{\pi_{1}}\approx {\langle \ell_{t-1}|y_{t}=1\rangle }_{t}$

$$\begin{array}{c} \text{log}\frac{\pi_{1}}{\pi_{0}}\approx \text{log}\pi_{1}\approx -\text{log}{\left\langle \ell_{t-1}|y_{t}=1\right\rangle }_{t}\leq {\left\langle \text{log}\ell_{t-1}|y_{t}=1\right\rangle }_{t}, \end{array}$$

where the final inequality follows from the fact that log is concave and Jensen’s inequality (with equality holding when the variance of *ℓ*_*t*−1_ given *y*_*t*_ = 1 is zero). Thus, assuming the variance of the time elapsed between conditioned odors is small, we can estimate the bias *b* in the online setting with the updates:

$$\begin{array}{cc} b & \leftarrow b+\frac{1}{t}[\frac{1}{2}(1-y_{t}+\ell_{t-1}y_{t})w^{⊤}x_{t}-y_{t}\text{log}\ell_{t-1}-b]. \end{array}$$

Substituting these approximations into the offline update rules in Eq 2 yields our online algorithm (Algorithm 1).

In view of Jensen’s inequality, if the variance of the time elapsed between conditioned odors is large, then the bias *b* will be overestimated, meaning that the MBON will be less active than optimal. In other words, irregular intervals between DAN activity biases the MBON to be less active (i.e., predict that the unconditioned stimulus is present more often).

### Details of numerical experiments

The experiments were performed on an Apple iMac with a 3.2 GHz 8-Core Intel Xeon W processor. For each experiment, we used a learning rate of the form

$$\begin{array}{c} \eta_{t}=\frac{\eta_{0}}{1+\gamma t}. \end{array}$$

We chose the parameters *η*_0_ and *γ* by performing a grid search over *η*_0_ ∈ {1, 10^−1^, 10^−2^, 10^−3^, 10^−4^} and *γ* ∈ {10^−2^, 10^−3^, 10^−4^, 10^−5^, 10^−6^}. The optimal parameters for the synthetic dataset (resp. KC dataset) are *η*_0_ = 10^−1^ and *γ* = 10^−3^ (resp. *η*_0_ = 10^−1^ and *γ* = 10^−4^).

## Supporting information

S1 Appendix Comparison with a modified algorithm. We consider a modification of Algorithm 1 in which the DAN-induced plasticity of the KC-MBON synapses does not depend on the time elapsed since the last time the DAN was active.
(PDF)
