# Supplementary material for: A linear discriminant analysis model of imbalanced associative learning in the mushroom body compartment
Source: PLoS Comput Biol. 2023 Feb 6;19(2):e1010864. doi: 10.1371/journal.pcbi.1010864 (PMC9934445; doi:10.1371/journal.pcbi.1010864)
Supplement: S1 Appendix — We consider a modification of Algorithm 1 in which the DAN-induced plasticity of the KC-MBON synapses does not depend on the time elapsed since the last time the DAN was active. (PDF) [file pcbi.1010864.s001.pdf]

Comparison with a modified algorithm

We consider a modification of Algorithm 1 in which the DAN-induced plasticity of the KC-MBON synapses does not depend on the time elapsed since the last time the DAN was active. In particular, we modify Algorithm 1 so that ℓ_t is set to be identically ℓ_*. In other words, when $y_t = 1$, the update to the KC-MBON synapses in Eq 4 of the main text is replaced with

$$y_t = 1 : \qquad \Delta \mathbf{w} = -\eta \ell_* \mathbf{x}_t. \tag{1}$$

Along with the update in Eq 3 of the main text, these updates are closely related to the updates in equation 5 of [1]. We test this modified algorithm on the synthetic dataset with ℓ_* equal to 10 or 2, S1 Fig 1. As expected, in both cases the accuracy of the algorithm is optimal when $\ell_* = \frac{1}{\pi_1}$.

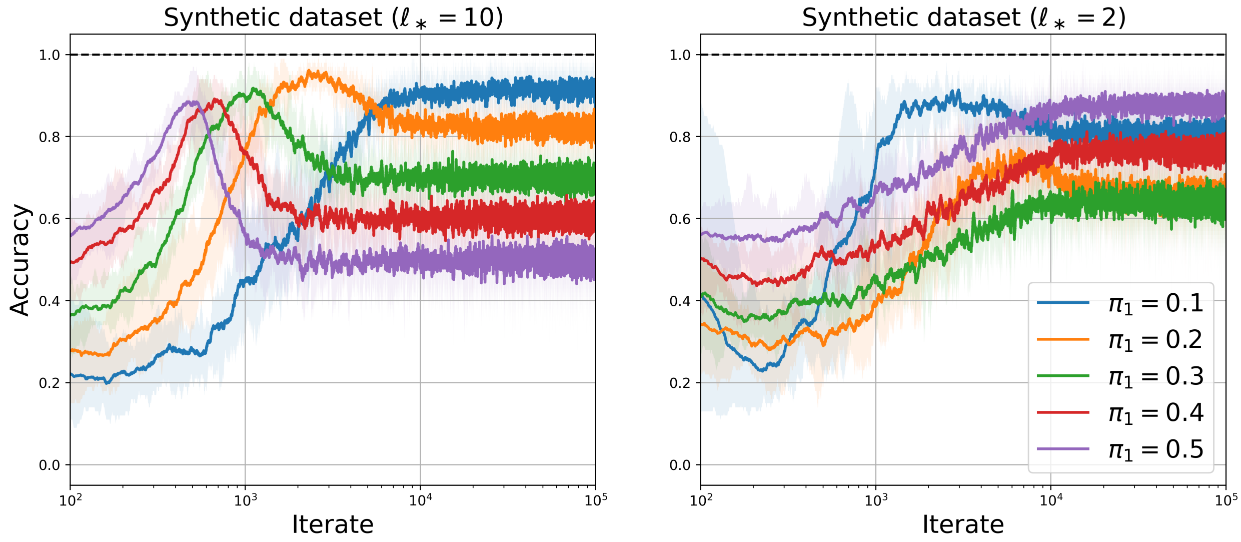

Figure 1: Performance of Algorithm 1 on the synthetic datasets with ℓ fixed. Each line denotes the mean accuracy over 10 runs. Each shaded region indicates the area between the minimum and maximum accuracy over 10 runs.

We also test the modified update rule in S1 Eq 1 on the KC dataset with competing MBONs, S1 Fig 2. Comparing the results with the performance of Algorithm 1, we see that the accuracy of both algorithms is high, suggesting that the KC representations are well separated so that even a non-optimal algorithm performs well. However, we see that when comparing odors 1 & 7, Algorithm 1 learns to accurately identify the odors much faster.

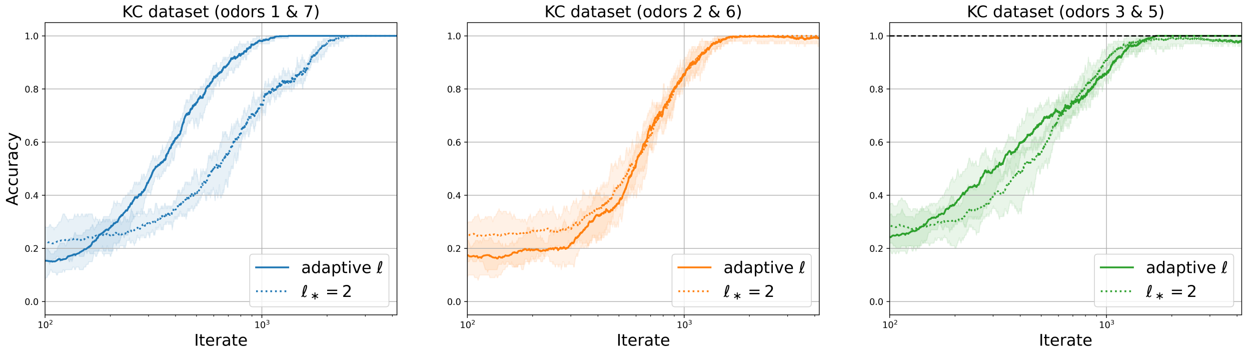

Figure 2: Parallel runs of Algorithm 1 (solid lines) or the modified algorithm with $\ell_* = 2$ (dotted lines) on the KC dataset with different class assignments (odors 1 & 7, 2 & 6 or 3 & 5) for each run. Each line denotes the mean accuracy over 10 runs. Each shaded region indicates the area between the minimum and maximum accuracy over 10 runs.

References

[1] James EM Bennett, Andrew Philippides, and Thomas Nowotny. Learning with reinforcement prediction errors in a model of the Drosophila mushroom body. *Nature Communications*, 12(1):1–14, 2021.
